# Association between Serum Concentrations of Apolipoprotein A-I (ApoA-I) and Alzheimer’s Disease: Systematic Review and Meta-Analysis

**DOI:** 10.3390/diagnostics11060984

**Published:** 2021-05-28

**Authors:** Marco Zuin, Carlo Cervellati, Alessandro Trentini, Angelina Passaro, Valentina Rosta, Francesca Zimetti, Giovanni Zuliani

**Affiliations:** 1 Department of Translational Medicine and for Romagna, University of Ferrara, 44121 Ferrara, Italy; marco.zuin@edu.unife.it (M.Z.); Angelina.passaro@unife.it (A.P.); valentinarosta89@gmail.com (V.R.); zlngnn@unife.it (G.Z.); 2 Department of Chemical, Pharmaceutical and Agricultural Sciences, University of Ferrara, 44121 Ferrara, Italy; Alessandro-trentini@unife.it; 3 Department of Food and Drug, University of Parma, 43121 Parma, Italy; francesca.zimetti@unipr.it

**Keywords:** ApoA1, Alzheimer’s disease, HDL

## Abstract

Background: A wealth of experimental and epidemiological evidence suggest that Apolipoprotein A-I (ApoA-I), the main protein constituent of high-density lipoprotein (HDL), may protect against Alzheimer disease (AD). To investigate this potential role, we conducted a meta-analysis of the published studies on the relationship between serum ApoA-I and AD occurrence. Methods: We screened MEDLINE, EMBASE, Web of Science, and Scopus, for cross-sectional studies published from inception to 1 March 2021, comparing the ApoA-I serum levels between patients with AD and cognitively normal controls. Results: From an initial screening of 245 articles, 5 studies, including 397 AD patients (mean age 75.0 years, 234 females) and 367 controls (mean age 69.2 years, 182 females), met the inclusion criteria. Compared to healthy controls, AD subjects had a lower ApoA-I serum level. The pooled weighted mean difference from a random-effects model was −0.31 g/L (*p* < 0.0001) (95% Confidence Interval: [−0.62–0.01], with high heterogeneity (I^2^ = 100%). The Egger’s test confirmed an absence of publication bias (t = 0.62, *p* = 0.576). Conclusions: Our study showed that AD patients present lower serum levels of ApoA-I compared to cognitively normal individuals. Further studies on large population samples are required to support this finding.

## 1. Introduction

Alzheimer disease (AD) is the most common cause of dementia in the elderly, accounting for more than two third of all cases [1]. Over the last decades, significant progress has been achieved in the understanding of the pathogenic mechanisms underlying this disease. The emerging picture is of a complex and multifactorial disease where the typical neuropathological hallmarks, represented by amyloid-β (Aβ) plaques and neurofibrillary tangles (NFT), are accompanied by other brain abnormalities that influence its clinical progression [2,3]. In particular, several evidences have been accumulating in support of a significant contribution of neurovascular dysfunction, already during the early stages of AD [4,5]. Indeed, ischemic and haemorrhagic stroke, one of the main and most severe clinical presentations of this cerebrovascular disease, has been often observed in patients with either pre-clinical or overt AD [6].

The main cause of stroke is doubtless represented by atherosclerosis, which is also associated with AD onset and progression. Therefore, the classical atherosclerosis risk factors are also good predictors of AD [7]. An emblematic example in this context is provided by high density lipoprotein cholesterol (HDL-C). A wealth of epidemiological evidence clearly suggests that the documented inverse association between its level and cardiovascular disease (CVD) may be driven by the biological function of HDL rather than the concentration of its cargo (Cholesterol) [8,9]. Accordingly, various components of the complex HDL proteome, the determinants of the functional proprieties of the lipoprotein, have been found to be strongly related with AD occurrence [10,11]. Among proteins, ApoA1 is essential for the biological activity of HDL, such as the ability to promote the reverse cholesterol transport process and to exert antioxidant and anti-inflammatory activities [10,12]. Intriguingly, this apolipoprotein is not synthetized in the brain as occurs for ApoE [13], but it is derived from the periphery, thanks to the ability to cross the brain blood barrier (BBB) through endocytosis processes [14,15] and localizes in the brain when it plays critical role in preserving cerebrovascular integrity. Mechanistically, apoA-I has shown an ability to influence Aβ deposition by preventing processing or favouring its clearance, as well as the capacity to mitigate oxidative stress and neuroinflammation [14].

A few number of studies report that AD patients present lower serum levels of apolipoprotein A1 (ApoA1), compared to cognitively normal individuals [14,16]. In addition, apoA-I levels were shown to correlate with disease severity [17]. However, these results were not confirmed by other studies [18], including a retrospective observation on postmenopausal women with early AD, in which serum ApoA-I levels were similar to those of control subjects [19]. 

Thus, given the limited data availability and the controversial results, in order to shed some light of the role of this HDL-associated protein in AD pathogenesis, the aim of the present manuscript is to provide a mini systematic review and meta-analysis analysing the available literature reporting the serum ApoA-I levels in patients with confirmed AD.

## 2. Materials and Methods

### 2.1. Data Sources

This study followed the Preferred Reporting Items for Systematic Reviews and Meta-analyses (PRISMA) reporting guidelines (Supplementary Table S1) [20]. We conducted a systematic literature search based on MEDLINE and Scopus, searching for all studies published from inception to 1 March 2021, comparing the APOA1 serum levels between patients with AD and cognitively normal controls. 

### 2.2. Data Extraction and Quality Assessment

The selection of studies to be included in our analysis was independently conducted by two authors (M.Z., A.T.) in a blinded fashion. Any discrepancies in study selection were resolved by consulting a third author (C.C.). The following MeSH terms were used for the search: “Alzheimer’s disease” AND “Apolipoprotein A-I” OR “ApoA-I”. Moreover, we searched the bibliographies of target studies for additional references. Case reports, review articles, abstracts, editorials/letters, and case series with less than 10 participants were excluded from the analysis. At the same manner, studies evaluating the role of ApoA-I serum levels in the progression of AD or studies without a control group were excluded. Data extraction was independently conducted by two authors (M.Z., G.Z.). For all studies reviewed, we extracted the mean age, female gender, mini-mental state examination (MMSE) value, the diagnostic criteria used for making the diagnosis of dementia, total cholesterol (TC), triglycerides (TG), high density lipoprotein (HDL), low density lipoprotein (LDL), and ApoA-I serum levels in both AD patients and controls. The quality of included studies was graded using the Newcastle-Ottawa quality assessment scale [21].

### 2.3. Data Synthesis and Analysis

Continuous variables were expressed as mean ± standard deviation (SD) while categorical variables as counts and percentages. Statistical analysis was performed using the weighted mean difference (WMD) methodology. Specifically, a random effect model presented with the corresponding 95% confidence interval (CI) was used to estimate the WMD pooled difference for APOA1 serum levels between AD patients and controls, using the same scale of measurement (g/L) for the outcome. Statistical heterogeneity was measured using the Higgins I^2^ statistic. A I^2^ = 0 was considered to indicate no heterogeneity, values of I^2^ as <25%, 25–75%, and above 75% to indicate low, moderate, and high degrees of heterogeneity, respectively [22]. To evaluate publication bias, both Egger’s test and funnel plots were computed. Meta-regression analysis using age and gender as moderator variables was also performed. All analyses were carried out using Review Manager 5.2 (The Cochrane Collaboration, Oxford, UK) and Comprehensive Meta-Analysis software, version 3 (Biostat Inc., Englewood, NJ, USA).

## 3. Results

### 3.1. Literature Search and Characteristics of Studies Included

**Figure 1:**
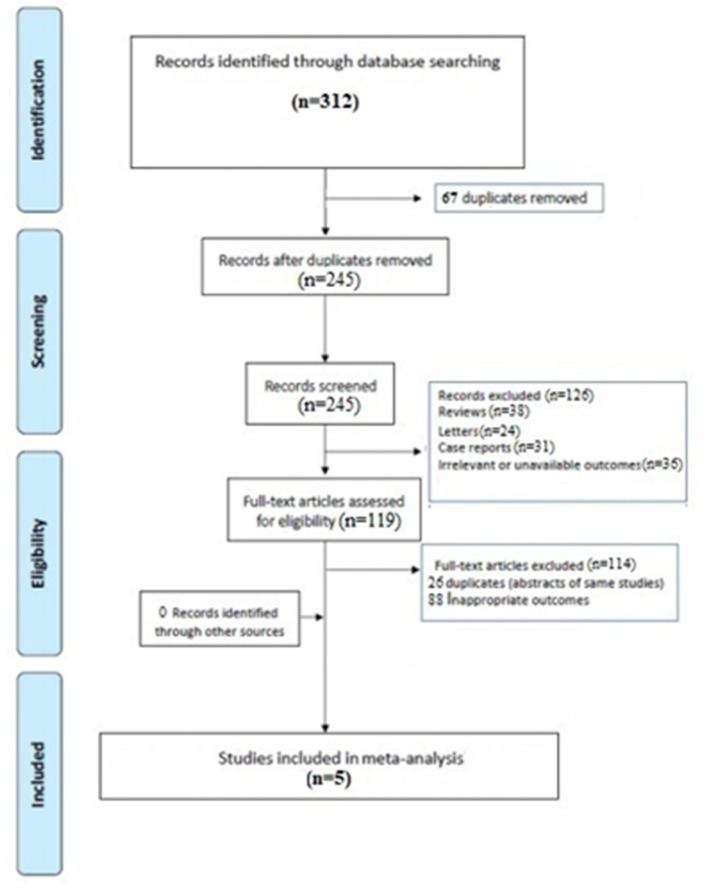
PRIMA flow chart.

A total of 245 articles were retrieved after excluding duplicates. Subsequently, 126 were excluded for not meeting the inclusion criteria, leaving 119 full-text articles to assess for eligibility. Finally, five articles met the inclusion criteria and were considered for the analysis [23,24,25,26,27]. (Figure 1).

The pooled subjects included a total of 397 AD patients (mean age 75.0 years, 234 females) and 367 controls (mean age 69.2 years, 182 females). As expectable, in the four studies reporting the results of the mini-mental state examination (MMSE) test, AD patients had a significantly lower score value (*p* < 0.001) compared to controls [24,25,26,27]. Four investigations used the NINCDS-ADRDA criteria for the diagnosis of dementia [23,25,26,27], while DSM criteria were also used in three studies as complementary diagnostic tool [23,24,26]. All Investigations resulted to be of moderate/high quality according to the NOS (Table 1). Three analysis reported the lipid profile of the patients enrolled and the relative values are shown in Table 2 [23,25,26].

### 3.2. ApoA1 Serum Levels between AD Cases and Controls

**Figure 2:**
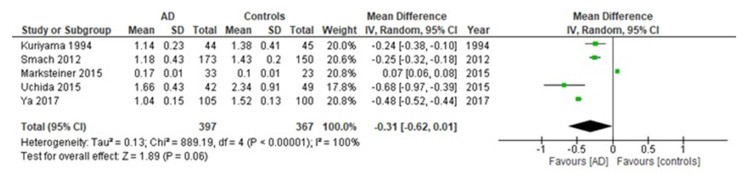
Forest plots for ApoA1 serum levels in AD and healthy controls.

**Figure 3:**
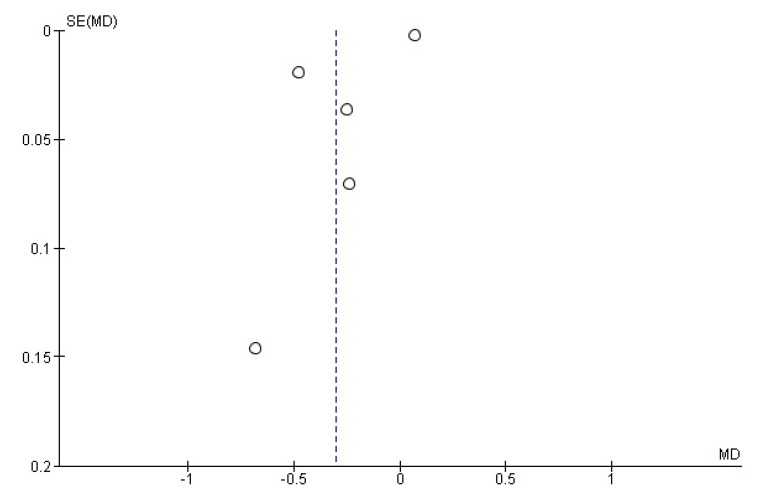
Funnel plots for ApoA1 levels in AD and healthy controls in included studies. Vertical dashed lines represent the summary weighted mean difference (*WMD*).

Compared to healthy controls, AD subjects had a significantly lower ApoA1 serum level. The pooled WMD from a random-effects model was −0.31 g/L (*p* < 0.0001) (95% CI: [−0.62–0.01]; I^2^ = 100%; test for overall effect: Z = 1.89; *p* =0.06 (Figure 2). The Egger’s test confirmed the absence of publication bias (t = 0.62, *p* = 0.576). The relative funnel plot is shown in Figure 3.

### 3.3. Meta-Regression

Meta-regression analysis revealed a direct association between ApoA-1 serum levels [Coeff. 0.745, Standard Error—SE—0.219, 95% CI: 0.315–1.175, *p* = 0.0007] and female gender [Coeff. 0.648, SE 0.209, 95% CI: 0.237–1.059, *p* = 0.002.]

## 4. Discussion

The purpose of this study was to investigate the relationship between serum ApoA-I levels and AD. This first metanalysis on this issue showed that patients with AD present a significantly lower level of serum ApoA-I compared to cognitively normal individuals.

This apoprotein plays a pivotal role in the reverse cholesterol transport, the atheroprotective process mediating the removal of excess cholesterol from peripheral tissues to the liver for the final excretion through the bile [28,29]. Indeed, it is directly implicated in the interaction of HDL with the ATP-binding cassette transporter A1 (ABCA1) and the enzyme lecithin cholesterol acyltransferase (LCAT), responsible for cholesterol efflux from macrophages and cholesterol esterification (and maturation of HDL), respectively [30]. Besides, ApoA-I contributes, along with other so-called accessory proteins, such as paraoxonase 1 (PON1) and lipoprotein lipase A2, to the HDL anti-inflammatory and antioxidant activity [14]. All these actions make ApoA1 the main determinant of the well-known antiatherogenic propriety of HDL. As atherosclerosis is a widely recognized risk factor for AD, it is conceivable to hypothesize that ApoA-I could also protect from AD.

The protection of ApoA1 from this neurodegenerative disease could not be a mere reflection of its beneficial systemic effect. Indeed, Apo A-I has been demonstrated to cross the BBB through a transcytosis process [14] independent of clathrin, but mediated by the HDL receptor SR-BI [31], or, alternatively, through an involvement of the LDL receptor-related protein family [32].

Consistently, the ApoA-I mimetic peptide 4F was able to efficiently cross the BBB after systemic injection in mice [33].

However, the definitive supportive evidence on this HDL transfer in humans is still lacking and further investigations are needed. On the contrary, it is well-established that the ApoA-I present within the brain parenchyma and CSF comes from periphery and its level correlates with its respective in plasma circulation [34]. A wealth of in vitro and in vivo evidence suggests that brain ApoA-I may be implicated in the AD pathogenic mechanism. Indeed, it can inhibit aggregation and promote clearance of Aβ, thus affecting the formation of neuritic plaques. Intriguingly, in this context, animal models of AD overexpressing human ApoA-I were characterized by an improvement of memory deficit and attenuation of Aβ-associated neuroinflammation [35], while ApoA-I deletion exacerbates cerebral Aβ deposition and astrocyte activation in mice [36]. Interestingly, the capacity of ApoA-I to promote the clearance of Aβ seems to be dependent on its lipidation degree, reaching the maximal efficiency with ApoAI-discoidal particles [37].

Human studies also support this potential protective role of ApoA-I against AD. Slot et al. found that lower plasma ApoA-I levels in 429 non-demented individuals are linked with increased risk of developing the disease [16]. Moreover, this study along with a more recent one found that peripheral ApoA-I is inversely associated with biomarkers of NFT pathology, such as t-tau and p-tau. In addition, plasma levels of ApoA-I have been found to inversely correlate with cognitive performances and disease severity (Merched et al., 2000). Consistently, lower plasma levels of ApoA-I have been associated with a reduction of hippocampal and whole brain volume and cortical thickness in AD patients (Hye et al., 2014). In apparent contradiction, three meta-analyses failed to show an association between HDL-C levels and AD risk [38,39,40]. However, this is not surprising, because the HDL-C levels only partly determine HDLs biological functionality. In this regard, it is worth mentioning that an impairment of both plasma HDL and isolated apoA-I cholesterol efflux-promoting function has been previously observed in AD patients, correlating with the degree of the cognitive decline [41]. Unfortunately, as highlighted by our analysis, previous investigations have rarely analyzed the relationship between apoA-I and AD, as demonstrated by the few studies reporting such data. Therefore, our preliminary findings should be considered the basis for further analyses on this issue.

Cumulating epidemiological evidence suggests that targeting the pleiotropic nature of HDL, instead of the concentration of the carried cholesterol, may represent the best therapeutic approach against atherosclerosis and related diseases [42,43]. However, the clinical trials using recombinant ApoA-1 infusions that have been undertaken starting from this rationale failed to provide positive outcomes [44]. One of the possible explanations of this failure may lie in the complex structural and functional nature of HDL proteome [14,45,46]. Indeed, the above-mentioned accessory proteins chiefly influence ApoA-1 activity; thus, increasing the level of this apoprotein may be ineffective if not accompanied by a concomitant improvement of the other proteome components. To the best of our knowledge, no clear evidence of a positive effect of ApoA-1 or HDL-C therapy on AD patients has been collected so far. On the contrary, drugs commonly used for treatment and prevention of hypercholesterolemia (in primis, statins) atrial fibrillation (e.g., antiocoaugulants and antiplatelets) yield promising results in the in the context of dementia [47,48,49,50,51].

Our analysis has some limitations. Firstly, being based on observational studies, the possibility of remaining residual confounding due either to unmeasured or underestimated risk factors in the reviewed studies cannot be excluded, representing a potential source of biases. This aspect seems to be confirmed by the high heterogeneity observed in our results. This could be, at least partially, explained by the strong influence of female gender on both ApoA1 levels and AD incidence and prevalence. Moreover, very few investigations, enrolling few AD patients, focused their attention on the APOA1 serum levels, limiting the number of studies included and as consequence also our conclusions. The absence of correction for potential confounding factors in the original analysis causes us to take our preliminary results cautiously. In the same manner, since only three investigations reported the HDL-C levels, a potential meta-regression using this variable as moderator resulted in being impossible.

## 5. Conclusions

In conclusion, our study showed that AD patients present lower serum levels of ApoA-I compared to cognitively normal individuals. Further studies conducted on larger population samples are mandatory to support this finding.

## Figures and Tables

**Table 1:** General characteristics of the population enrolled.

Authors	Type of Manuscript	AD (n)	Controls (n)	AD Patients		Controls		MMSE Score		Diagnostic Criteria	NOS
				**Mean Age**	**Females** **(%)**	**Mean Age**	**Females** **(%)**	**AD**	**Controls**		
Kuriyama 1994 [23]	Case-control	44	45	75.4 ± 6.0	37 (84.0)	69.6 ± 8.9	27 (60.0)	NR	NR	DSM-III-R NINCDS-ADRDA	8
Smach 2012 [25]	Case-control	173	150	75.0 ± 1.8	99 (57.2)	71.1 ± 1.7	79 (52.6)	15.0 ± 1.8	28.0 ± 0.32 **	NINCDS-ADRDA	8
Uchida 2015 [24]	Case-control	42	49	73.9 ± 7.4	32 (76.1)	69.8 ± 12.4	16 (32.6)	18.3 ± 5.8	28.8 ± 1.6 **	DSM IV	7
Ya 2017 [26]	Case-control	105	100	69.9 ± 4.4	45 (42.8)	64.8 ± 5.8	50 (50)	17.3 ± 5.5	26.8 ± 4.7 **	DSM-IV-R NINCDS-ADRDA	8
Marksteiner 2015 [27]	Case-control	33	23	81.0 ± 1.0	21 (63.6)	71.0 ± 1.4	10 (43.4)	20. 0± 0.8	29.0 ± 0.2 **	NINCDS-ADRDA	7

AD: Alzheimer’s disease; NR: Not reported; NINCDS-ADRDA: National Institute of Neurological and Communicative Disorders and Stroke and the Alzheimer’s Disease and Related Disorders Association criteria; DSM: Diagnostic and Statistical Manual of Mental Disorders; NOS: Newcastle-Ottawa quality assessment scale. ** *p* < 0.001.

**Table 2:** Lipid profile of the population analysed.

Authors	Total Cholesterol (mmol/L)		Triglycerides (mmol/L)		HDL-C (mmol/L)		LDL-C (mmol/L)	
	**AD**	**Controls**	**AD**	**Controls**	**AD**	**Controls**	**AD**	**Controls**
Kuriyama 1994 [23]	4.75 ± 1.14	4.96 ± 0.87 **	1.11 ± 0.47	1.16 ± 0.58	1.1 ± 0.43	1.38 ± 0.27 **	3.14 ± 1.00	3.1 ± 0.75
Smach 2012 [25]	4.86 ± 1.11	4.58 ± 1.21	1.19 ± 0.7	1.59 ± 0.76 **	0.82 ± 0.22	1.23 ± 0.29 **	3.47 ± 0.9	3.24 ± 0.91
Uchida 2015 [24]	NR	NR	NR	NR	NR	NR	NR	NR
Ya 2017 [26]	4.35 ± 0.17	4.76 ± 0.09	1.21 ± 0.15	1.48 ± 0.12	1.95 ± 0.26	1.37 ± 0.18 *	2.76 ± 0.25	2.84 ± 0.21
Marksteiner 2015 [27]	NR	NR	NR	NR	NR	NR	NR	NR

NR: Not reported: HDL: High density lipoproteins; LDL: Low density lipoprotein. * *p* < 0.05, ** *p* < 0.001. Data expressed in mg/dl in the original studies and converted to mmol/L for homogeneity.

## Data Availability

Not applicable.

## Supplementary Materials

The following are available online at https://www.mdpi.com/article/10.3390/diagnostics11060984/s1, Table S1: Prisma flow-chart.
